# Plasmons Enable Ultralow Threshold Solid-State Triplet Fusion Upconversion with a 2D Sensitizer

**DOI:** 10.1021/acs.nanolett.6c00279

**Published:** 2026-03-16

**Authors:** Seamus S. Lowe, Satya P. Butler, John E. Anthony, Saien Xie, Barry P. Rand

**Affiliations:** † Department of Mechanical and Aerospace Engineering, 6740 Princeton University, Princeton, New Jersey 08544, United States; ‡ Department of Chemistry, 4530 University of Kentucky, Lexington, Kentucky 40506, United States; § Department of Electrical and Computer Engineering, Princeton University, Princeton, New Jersey 08544, United States; ∥ Princeton Materials Institute, Princeton University, Princeton, New Jersey 08544, United States; ⊥ Andlinger Center for Energy and the Environment, Princeton University, Princeton, New Jersey 08544, United States

**Keywords:** upconversion, plasmons, 2D materials, interfaces, TMD, excitons

## Abstract

Solid-state triplet–triplet annihilation (TTA) upconversion has significant potential for application in light harvesting, optoelectronic devices, and bioimaging. However, the high optical powers required to achieve efficient upconversion have inhibited its adoption. In this work, we demonstrate plasmon-enhanced near-infrared (NIR)-to-blue TTA upconversion in a monolayer WSe_2_/organic heterojunction. Under far-field excitation, the device reaches a threshold of 19 mW/cm^2^ and an external quantum efficiency (EQE) of 0.17% with an anti-Stokes shift of 1.1 eV. Plasmon excitation lowers the threshold to 0.9 mW/cm^2^ and improves the EQE to 3.6%. We attribute the plasmon enhancement to surface plasmon polariton (SPP) near-field enhancement and dark-exciton absorption. Optimization of the WSe_2_ transfer process is identified as a key factor for the device performance. This work demonstrates that plasmon excitation overcomes the low far-field absorption of 2D transition-metal dichalcogenide (TMD) sensitizers. Consequently, monolayer TMDs can achieve solid-state upconversion with a performance among the best reported.

## Graphical abstract

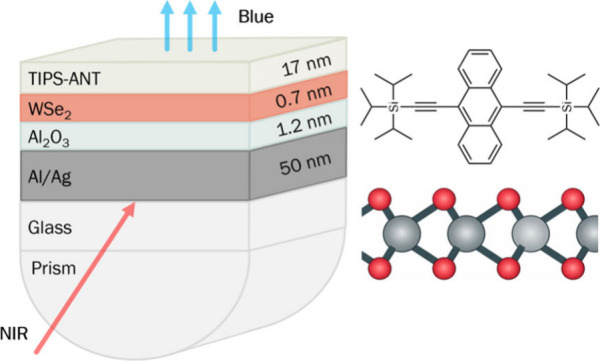

Solid-state triplet–triplet annihilation (TTA) upconversion has potential applications in solar cells, photoredox catalysis, light-emitting devices, and bioimaging. Holding back such applications has been the high optical powers (typically hundreds to thousands of mW/cm^2^) required to achieve maximum upconversion efficiency. Monolayer transition-metal dichalcogenides (TMDs) are a class of 2D materials emerging as promising semiconductor materials for a wide array of applications. They offer several unique advantages as sensitizers for thin-film upconversion devices. In thin-film devices, TTA upconversion is often diffusion-limited because triplet excitons need to reach an interface to transfer between a sensitizer and an annihilator. In 2D materials, charge carriers are restricted to 2 degrees of freedom. Consequently, all excitons in a TMD monolayer covered with organic molecules bypass the exciton diffusion bottlenecks. However, although monolayer TMDs exhibit high oscillator strengths, single monolayers absorb only a small fraction of incident light. To overcome this obstacle, we explored the use of surface plasmon polaritons (SPPs) to excite excitons in the TMD monolayer as opposed to far-field irradiation. The extreme electromagnetic confinement of SPPs creates a strong field that decays evanescently over 10–20 nm. The absorption of subnanometer-thick TMD monolayers that can easily sit within this strong field will be significantly enhanced, making these 2D semiconductors very interesting for upconversion. Additionally, the strong out-of-plane field component and in-plane momentum enable direct absorption by dark excitons, which are inaccessible to far-field photons. This increases the intrinsic absorption of a TMD monolayer.

Previous literature has demonstrated TTA upconversion with monolayer TMDs as a sensitizer under far-field irradiation.^1,2^ Additionally, enhanced absorption of monolayer WS_2_ with plasmons has been observed.^3^ Recent work with upconversion in organic thin films has also shown that SPPs can significantly decrease the upconversion threshold intensity compared to far-field excitation.^4^ This work presents a device stack comprising a TMD/organic heterojunction placed on a silver thin film separated by a thin alumina spacer. The heterojunction consists of a tungsten diselenide (WSe_2_) monolayer and a 17-nm-thick neat film of 9,10-bis­[(triisopropylsilyl)­ethynyl]­anthracene (TIPS-ANT). This device exhibits anti-Stokes emission under far-field irradiation with an upconversion threshold of 19.0 mW/cm^2^—several times lower than previously demonstrated in TMD/organic heterojunctions—and an external quantum efficiency (EQE) of 0.17%. By exciting with near-infrared (NIR) light at 730 nm and collecting blue emission up to 445 nm, this device achieves an anti-Stokes shift of 1.1 eV. Using a Kretschmann excitation configuration (i.e., using a prism) to couple to SPPs in the silver film, an upconversion threshold of 0.9 mW/cm^2^ and an EQE of approximately 3.6% were achieved, among the best reported values for solid-state TTA upconversion.

For this study, we use monolayer WSe_2_ as a sensitizer grown by metal–organic chemical vapor deposition (MOCVD).^5^ While the previous report of WSe_2_ as a sensitizer for photonic upconversion used mechanically exfoliated flakes to fabricate heterojunctions,^1^ MOCVD allows for the growth of larger uniform flakes with high coverage enabling scaled application. For this work, large flakes on the order of 30 μm were grown to produce a 1 × 1 cm film (Figure 1a). To verify that grown WSe_2_ was a monolayer in nature, Raman spectroscopy and photoluminescence (PL) measurements were performed (Figure 1c,d). These align with reported observations for WSe_2_ monolayers, with the major E′ Raman mode dominating near 250 cm^–1^ and a single PL emission peak near 745 nm.^6^ Monolayer WSe_2_ is a direct-band-gap semiconductor that, when optically excited, hosts excitons of a hybrid Wannier–Mott (i.e., delocalized) and Frenkel (i.e., localized) character with a typical Bohr radius of 1.5 nm and large binding energies of 200–700 meV.^7,8^ For WSe_2_, the lowest-energy excitonic transition lies in the K valley of the Brillouin zone with an energy of 1.65–1.75 eV. However, the conduction and valence bands (CB and VB, respectively) at the K valley are spin split due to strong spin–orbit coupling (SOC) in the monolayer. As shown in Figure 1e, the VB experiences a large splitting while the CB is split by only 30–50 meV. Because the lower CB and upper VB have opposite spins, this lowest-energy transition is optically forbidden, or a “dark” exciton. The optically allowed transition, or “bright” exciton, is from the upper VB to the upper CB and is therefore 30–50 meV higher in energy than the dark transition. The strong SOC and exchange interaction present in monolayers allows for significant mixing of bright and dark excitonic states. An important feature is that the transition dipole for bright excitons is oriented in-plane with respect to the monolayer while the dominant dipole for dark excitons is generally out-of-plane (Figure 1b).^9^

**Figure 1:**
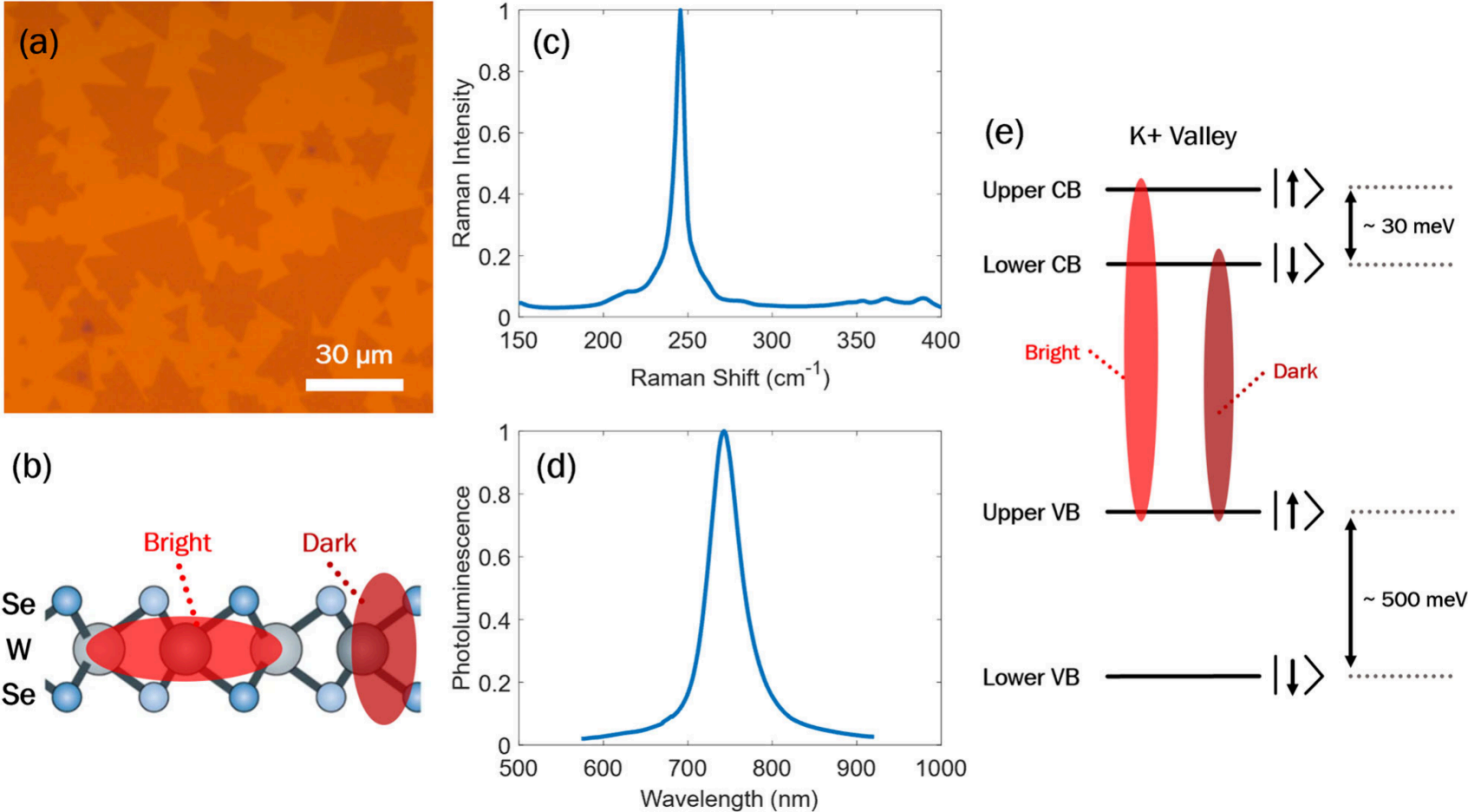
(a) Optical microscopy image of monolayer WSe_2_ domains. (b) Diagram showing the orientation of “bright”, or in-plane, A-excitons and “dark”, or out-of-plane, A-excitons in the WSe_2_ monolayer. Note that excitons are not drawn to scale. (c) Raman spectrum of the WSe_2_ domains. (d) PL spectrum of the WSe_2_ domains. (e) Diagram of the band splitting at the K^+^ valley in monolayer WSe_2_ with spin-allowed bright and spin-forbidden dark excitons. The K^–^ valley experiences the same splitting with opposite spin directions.

To achieve upconversion using monolayer WSe_2_ as a sensitizer, TIPS-ANT (Figure 2a) was selected as an organic annihilator. With triplet and singlet energies of approximately 1.4 eV (energetically downhill from the WSe_2_ A-exciton at 1.7 eV) and 2.7 eV, respectively, TIPS-ANT can realize a large anti-Stokes shifted emission as shown in the energy-level diagram in Figure 2c. A bilayer upconversion film was tested to demonstrate upconversion at the WSe_2_/TIPS-ANT heterojunction, as schematically shown in Figure 2b. To do so, WSe_2_ flakes were grown on Si/SiO_2_ and then transferred to silver/alumina (Al_2_O_3_) thin films (Figure 2b). The alumina cap serves as a spacer between the WSe_2_ monolayer and the silver substrate to prevent quenching by the metal film. A neat 17-nm-thick film of TIPS-ANT was then evaporated onto the WSe_2_ sensitizer. The stack was gently annealed in a N_2_ glovebox. It has been observed that even moderate strain can significantly shift the A-exciton absorption peak in monolayer TMDs.^10^ Annealing monolayers in an inert environment has been demonstrated as an effective method to relieve strain.^11^ Gentle annealing may also allow for the desorption of surface contaminants and improve the interfacial contact between the monolayer and organic films. Figure 2d shows spectra for the steady-state PL of WSe_2_ pumped at 532 nm and of TIPS-ANT pumped at 375 nm and the spectra of upconverted emission from the heterojunction pumped at 730 nm. The steady-state absorption of WSe_2_ is difficult to measure directly on a silver/alumina substrate, but the absorption profile of the A-exciton is similar to the A-exciton PL peak, although it may be somewhat narrower and blue-shifted.^1^ In this bilayer device, 730 nm excitation populates excitons in the WSe_2_ monolayer, which transfer across the heterojunction interface to triplet excitons in TIPS-ANT. The dominant mechanism of exciton transfer from monolayer TMDs to neighboring organic films has been shown to be either one-step Dexter energy transfer (DET) or two-step indirect triplet energy transfer (TET).^12^ Following interfacial transfer, triplet excitons diffuse and annihilate to form high-energy singlet excitons in TIPS-ANT, resulting in blue emission up to 445 nm. A control experiment exciting a neat film of TIPS-ANT without WSe_2_ yielded no anti-Stokes emission.

**Figure 2:**
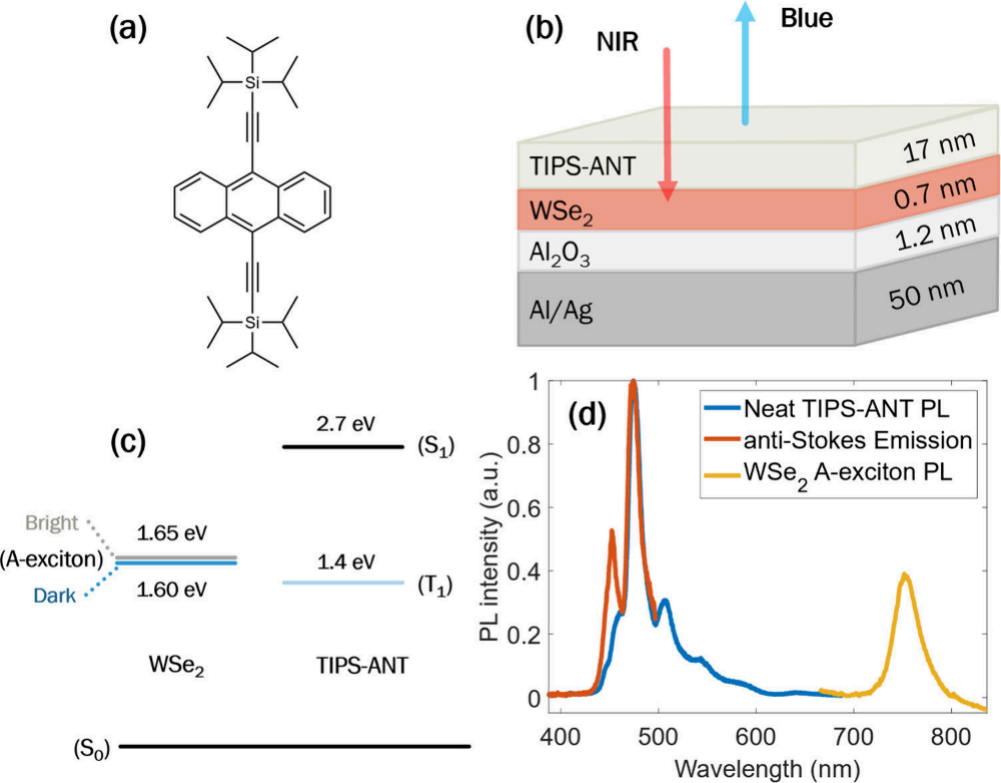
(a) Molecular structure of TIPS-ANT. (b) Schematic of the heterojunction for far-field-excited NIR-to-blue upconversion. (c) Energy-level diagram showing energies of bright and dark A-excitons in WSe_2_ and singlet and triplet excitons in TIPS-ANT. (d) Spectra of neat TIPS-ANT emission, upconverted emission from TIPS-ANT, and A-exciton emission of the WSe_2_ monolayer in the heterojunction.

The TMD transfer process uses a spin-coated polycarbonate (PC) overlayer to effectively lift WSe_2_ flakes off the growth substrate and move them onto the target substrate with minimal damage, as shown in Figure 3a. Thermal release tape (TRT) is used to lift the TMD/PC stack off the substrate and place it on the target substrate. When removed from the growth substrate, water is allowed to intercalate under the TMD to assist lift-off. After the transfer, the PC layer is dissolved with a chloroform (CF) bath and isopropyl alcohol (IPA) rinse. The post-transfer surface quality of WSe_2_ becomes a critical factor for the upconversion performance because intimate contact across the TMD/organic interface facilitates excitation of triplet excitons in TIPS-ANT. The efficiency of this transfer process, whether dominated by DET or TET, falls off sharply with distance. Consequently, any polymer residue at the interface will increase the distance between WSe_2_ and TIPS-ANT, inhibiting exciton transfer. This will be particularly true for DET, which requires wave function overlap. To ensure a clean interface, the PC backbone was dissolved in a CF bath for 2 h, washed with IPA, and gently annealed in N_2_. To quantify the remaining PC on the surface, Figure 3b shows the intensity of the carbon 1s (C 1s) signal on a silicon substrate from X-ray photoelectron spectroscopy (XPS) as a function of the CF dissolution time at room temperature. After 2 h of dissolution time, the C 1s signal reaches the baseline intensity. This is the same C 1s signal intensity measured for a sample without a carbon-based overlayer and which sources from adventitious carbon as a result of atmospheric exposure.

**Figure 3:**
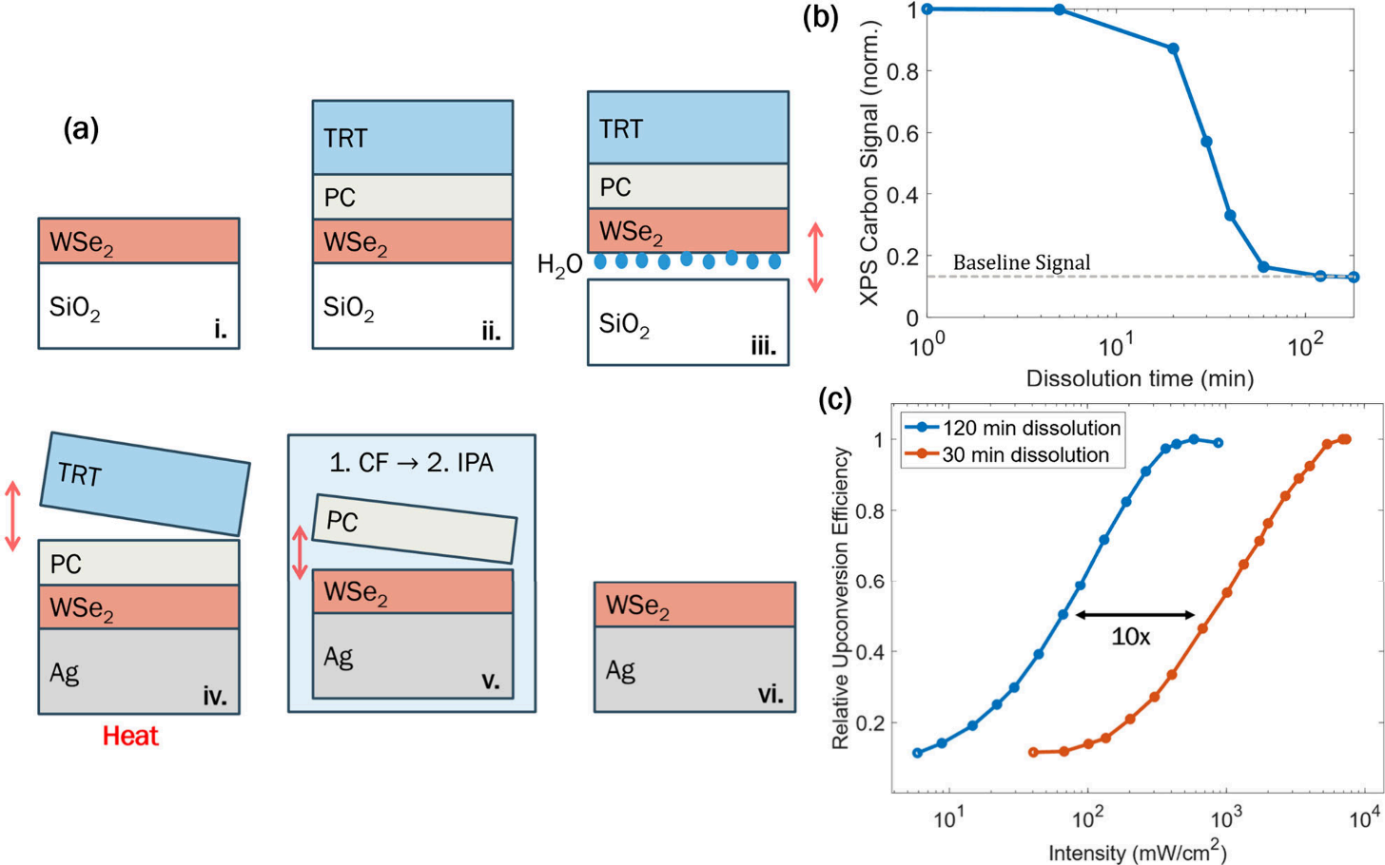
(a) TMD transfer process from growth substrate (SiO_2_) to target substrate (Ag) using TRT coated with PC and immersion in CF and IPA baths. (b) XPS carbon signal as a function of the dissolution time of the PC overlayer. (c) Upconversion performance, relative efficiency vs excitation intensity, for heterojunctions where the PC overlayer has been dissolved for 30 vs 120 min.

The impact of the surface quality on upconversion is exemplified in Figure 3c by comparing power-dependent upconversion for two heterojunctions with different degrees of surface purity. For a 2 h CF dissolution of PC, the film exhibited an upconversion threshold of 19 mW/cm^2^. With a decrease in the time of PC dissolution to 30 min, the upconversion threshold intensity increased by approximately an order of magnitude to 198 mW/cm^2^. Thus, the upconversion performance serves as an indirect quantifier of the TMD surface quality in this system.

To excite the heterojunction with SPPs, the sample was attached to a half-cylinder fused silica prism by using index matching oil to create a Kretschmann excitation configuration (Figure 4a). In this geometry, the excitation laser enters through the prism and reflects off the silver film. The angle of incidence can be swept to modify the in-plane momentum, *k*, of the incoming photons. At the necessary in-plane momentum, TM-polarized (or p-polarized) photons will resonantly couple to SPP modes. The intensity of the reflected TM light is plotted against the angle of incidence (Figure 4b) to locate the plasmon resonance angle. A dip in the intensity is clearly resolved, reaching a minimum of 33% reflectance at 54° from normal incidence. At this angle, the reflectance minimum indicates that a maximum number of incoming TM photons (67%) are coupling to plasmon modes, creating SPPs on the metal surface. To further characterize the surface plasmon dispersion in this device, ellipsometry was used to map the p-polarized reflectance as a function of the photon energy and *k* (Figure 4c). This provides the eigenenergies of the plasmon modes for a given *k*. The resulting map depicts the dispersion curve for surface plasmons, and at a photon energy of 730 nm (1.70 eV), the corresponding angle from the dispersion curve is indeed 54° from normal. The continuous dispersion curve also shows no avoided crossing at the A-exciton energy (near 1.7 eV), indicating no strong coupling between A-excitons and SPPs despite resonant excitation at the A-exciton absorption.

**Figure 4:**
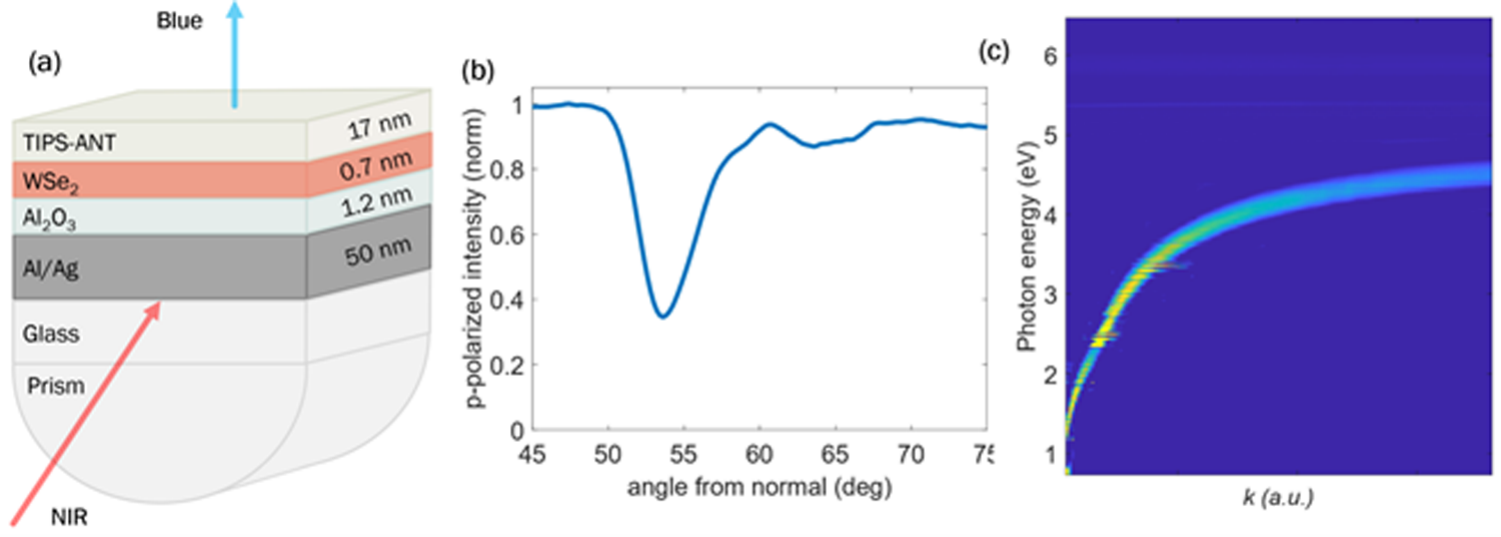
(a) Device schematic of the plasmon-enhanced upconversion film and Kretschmann excitation geometry. (b) Reflectance dip of the plasmon resonance angle with 730 nm excitation. (c) Plasmon dispersion mapped with p-polarized reflectance as a function of the photon energy and angle of incidence from normal (photon in-plane momentum).

To quantify the performance of the far-field- and plasmon-excited upconversion films, power-dependent upconversion measurements were performed to obtain the upconversion threshold for each system. The optical power density is defined as the laser power divided by the beam area obtained from the Gaussian beam diameter. The laser power is measured with a photodiode power sensor, and the beam profile is captured with a CMOS camera. The relative upconversion efficiency is calculated by integrating the upconversion emission spectra, producing the brightness of anti-Stokes emission, and dividing by the respective optical excitation power. A greater brightness of anti-Stokes emission at a given power corresponds to a greater relative efficiency of upconversion. Figure 5a plots the relative efficiency against the corresponding excitation intensities for both far-field- and plasmon-excited devices. Under far-field excitation, the quadratic, linear, and saturation regimes emerge as the laser intensity is increased. The threshold intensity for upconversion corresponds to the excitation power at which the relative efficiency is 50% of the maximum. Under plasmon excitation, the intensities required to populate the full quadratic regime were too low to be reached because a Gaussian profile could not be reliably produced for larger beam diameters. Nevertheless, the threshold is still resolved by reaching intensities corresponding to less than 50% of the maximum. From Figure 5a, the far-field threshold intensity is identified as 19.0 mW/cm^2^ and the plasmon excited threshold as 0.9 mW/cm^2^. This corresponds to a 21.1× decrease in the threshold intensity when driving upconversion with surface plasmons as opposed to far-field excitation. At higher excitation intensities, the relative upconversion efficiency exhibits a roll-off that is commonly observed in solid-state TTA upconversion systems. This behavior is consistent with high-fluence saturation and loss processes, including triplet-charge annihilation, singlet fission in the annihilator, saturation of interfacial energy transfer, energy back-transfer, and other pathways, becoming significant.^13,14^ To study the efficiency of upconversion in these devices, an integrating sphere was used to calculate the EQE of the far-field-excited upconversion film. The upconversion EQE is defined as the number of emitted upconverted photons divided by the total number of excitation photons incident on the device. By correcting for the power difference used to drive the upconversion against the captured laser profile and extrapolating the upconversion emission profile cut off by the 495 nm short pass filter, we obtained an EQE of 0.17% for the far-field-excited upconversion film. The optical power used to collect the upconverted photons was 196 mW/cm^2^, which operates the film at maximum efficiency. An integrating sphere could not be used to directly measure the EQE of plasmon-excited upconversion in the Kretschmann configuration. To determine the EQE of the near-field device, we compared the brightness of anti-Stokes emission for far-field- and plasmon-excited devices at an optical intensity of 50 mW/cm^2^, at which both films operate at similar relative efficiencies, in a setup schematically shown in Figure 5b (Figures S1 and S2). This setup allows us to assume identical collection, spectral window, illuminated area, and detector linearity and thus relate anti-Stokes brightness enhancement to the EQE enhancement. The brightness of anti-Stokes emission with plasmon excitation was 21× higher than the brightness of far-field emission (Figure S3). Therefore, we can conclude that the EQE of the plasmon-excited device is approximately 21× higher than that of the far-field-excited system (Figure 5c).^4^ This yields an EQE of 3.6% for the plasmon-excited system. Both the far-field- and plasmon-excited devices exhibit stable operation with less than a 1% decrease in the upconversion intensity when excited near the threshold power for 1 h.

**Figure 5:**
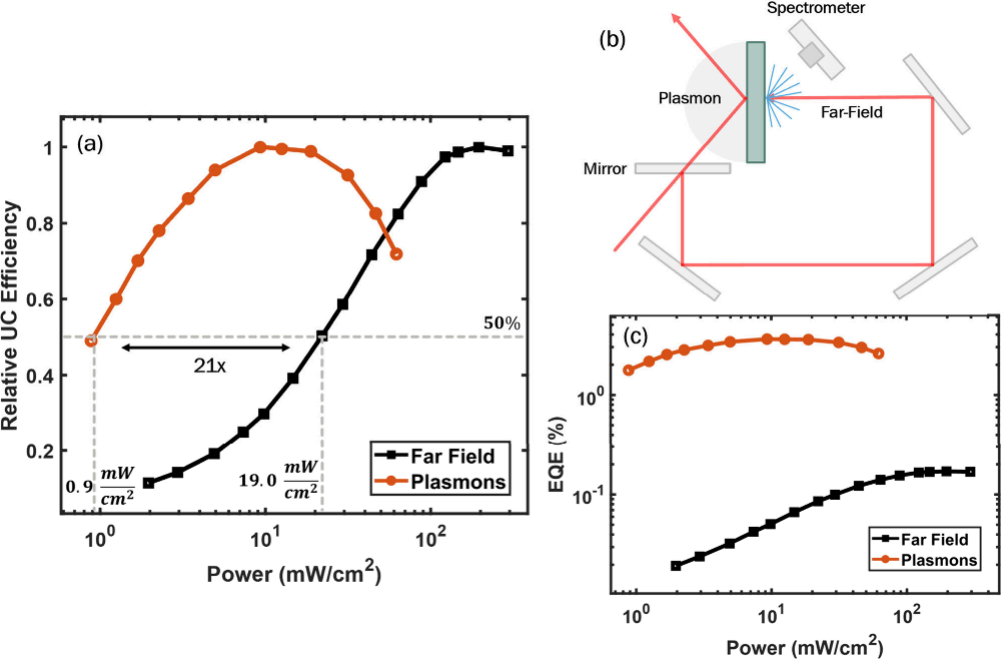
(a) Relative efficiency vs power density curves to identify plasmon and far-field excitation thresholds. (b) Schematic of the setup for comparing the upconversion intensities for plasmon and far-field excitation. (c) EQE as a function of the power density for plasmon and far-field excitation.

The major driver of the observed plasmon enhancement of upconversion is the large near-field intensity of the SPPs. With typical SPP confinements of 10–20 nm, the entire WSe_2_ monolayer situated 1.2 nm from the interface sits completely in the SPP hotspot. The absorption cross section of the monolayer is multiplied by the local field intensity enhancement. Thus, compared to far-field excitation, this results in more excitons being generated per unit of pump intensity assuming that population saturation is not reached. As a result, far lower pump intensities are required to reach the upconversion threshold, the point at which TTA starts to dominate over first-order triplet losses. Additionally, a greater proportion of incident photons will excite excitons in the TMD monolayer, which, assuming other loss channels remain unaffected, will boost the EQE by a similar degree.

Direct excitation of dark excitons is likely a further source of enhancement. It has been shown that dark excitons can be probed by outcoupling into SPP modes, allowing these states to be detected.^15^ This work reverses this process by directly exciting dark excitons with SPPs. Because these dark states are inaccessible to far-field photons, excitation with SPPs must necessarily increase the intrinsic absorption of the WSe_2_ monolayer relative to far-field excitation. These states are accessible to SPPs because they carry in-plane momentum well beyond the far-field light cone and have a strong out-of-plane field component. This allows for absorption from momentum-indirect states and states with out-of-plane transition dipoles, typically referred to as dark excitons. Dark excitons sit slightly lower in energy than bright A-exciton states and are longer-lived.^7^ The longer lifetime of dark excitons may result in a higher transfer yield to the annihilator relative to bright states.

The 1.2 nm alumina spacer thickness was selected to ensure a continuous pinhole-free dielectric barrier when grown with atomic layer deposition (ALD), effectively suppress direct-contact-induced charge/energy-transfer pathways with Ag, and allow the WSe_2_ monolayer to remain within the SPP near-field hotspot. Previous work has also shown that a spacer thickness of ∼1 nm between a metal film and WSe_2_ monolayer results in SPP excitation of a significant dark exciton population.^16^

Most existing solid films need tens to hundreds of mW/cm^2^ to reach the quadratic to linear threshold, *I*_th_, of TTA upconversion.^17^ Table 1 compares several leading TTA upconversion systems with the device developed in this work. Newer device architectures have recently pushed *I*_th_ under 10 mW/cm^2^ but operate with an EQE well under 0.1%. Modern heterojunction and bulk heterojunction (BHJ) devices have reported values of upconversion quantum efficiency, φ_UC_, in the 1–4% range.^18^ The EQE is approximately φ_UC_ multiplied by the efficiency of absorption and outcoupling. Based on these benchmarks, the presented plasmon-enhanced upconversion system exhibits performance metrics among the best reported, 0.9 mW/cm^2^ threshold and 3.6% EQE, along with one of the highest anti-Stokes shifts of 1.1 eV.

**Table 1:** Comparison of Upconversion Threshold and EQE/φ_UC_ for Several Leading TTA Upconversion Systems

sensitizer/annihilator	excitation to emission	EQE or φ_UC_	threshold, *I*_th_ (mW/cm^2^)	ref
TMD/organic film	NIR to blue	φ_UC_: 1.2%	110	Zhao et al. (2024)^1^
organic film	NIR to visible	φ_UC_: 4.0%	800	Sawa et al. (2023)^19^
perovskite/organic	NIR to visible	N/A	18	Sullivan et al. (2022)^20^
planar SPP-pumped organic film	green to blue	EQE: 0.094%	3.4	Wisch et al. (2026)^4^
organic BHJ	IR to visible	φ_UC_: 2.2%	10	Bi et al. (2024)^21^
crystalline organic heterojunction	green to blue	φ_UC_: <0.1%	0.8	Oldenberg et al. (2016)^22^
planar SPP-pumped TMD/organic film	NIR to blue	EQE: 3.6%	0.9	this work

A small number of upconversion devices based on a monolayer TMD/organic heterojunction have been previously demonstrated under far-field excitation.^1,2^ The lowest upconversion threshold previously achieved with such heterojunctions is 110 mW/cm^2^. Thus, the 19.0 mW/cm^2^ threshold achieved under far-field excitation by this work represents a 5.7× improvement over the existing standard. The 0.9 mW/cm^2^ threshold achieved under plasmon excitation extends this improvement to 120×.

This work demonstrates that SPP excitation makes 2D TMDs an exciting choice as a sensitizer for solid-state TTA upconversion, achieving a field-leading performance as a sensitizer with an organic annihilator. The SPP-driven WSe_2_/TIPS-ANT heterojunction exhibits a threshold of 0.9 mW/cm^2^ and an EQE of 3.6%. These metrics constitute a leap toward practical applicability for thin-film NIR-to-blue upconversion. Optimization of the TMD transfer procedure (enabling SPP excitation) is critical for the device performance. In addition to minimizing TMD strain and damage during transfer, the post-transfer TMD surface quality, dictated by dissolution of the polymer overlayer, is critical to achieving the maximum performance. In this work, MOCVD is utilized to achieve high coverage of large WSe_2_ flakes, enabling the fabrication of a centimeter-scale upconversion thin-film device.

While the threshold and EQE achieved in this work are indeed among the best reported for solid-state systems, several aspects of the demonstrated device could be optimized to further improve these. In the Kretschmann configuration, a 67% drop in TM-polarized light was observed, indicating 67% of incident photons coupling to SPPs. This could reasonably be increased to over 90% with more effective plasmon coupling, resulting in a stronger near-field and more efficient exciton generation (reducing threshold and boosting EQE). To achieve higher coupling, the thickness of the alumina spacer and the silver film could be further refined, and higher-quality and single-crystal silver would decrease plasmon losses. Additionally, while a high coverage of WSe_2_ flakes was utilized in this work, growth of a continuous monolayer WSe_2_ film would achieve perfect coverage and boost the upconversion performance.

Several aspects of this work require further study to fully understand the mechanism for the observed results. The precise mechanism of exciton transfer from WSe_2_ to triplets in TIPS-ANT is not fully understood. While recent work with WSe_2_ and rubrene has revealed one-step DET-mediated and two-step CT-mediated transfer as TMD-to-organic exciton-transfer mechanisms, a similar study should be conducted with a WSe_2_/TIPS-ANT heterojunction to determine which mechanism dominates in this system and whether those findings can be generalized.^12^ Furthermore, the relationship of bright and dark excitons to exciton-transfer kinetics and to the observed enhancement of SPP excitation is a particularly interesting avenue for further study.

Plasmon excitation of the monolayer TMD/organic heterojunction can also be extended to other choices of TMD sensitizers and organic annihilators. WS_2_ offers A-exciton absorption up to around 600 nm, while MoTe_2_ can push further into the NIR, absorbing down to near 1150 nm. Coupled with a growing library of organic annihilators from which to choose, the output energies can be readily tuned.

## Methods

Silver/alumina substrates were fabricated via physical vapor deposition using an Angstrom thermal evaporator with a base pressure of ∼1 × 10^–7^ Torr. A 1 nm seeding layer of aluminum was deposited on a glass substrate at 0.1 Å/s, followed by a 49 nm layer of silver at 1 Å/s. A 1.2 nm capping layer of Al_2_O_3_ was then grown with 15 cycles of atomic layer deposition (ALD) at 125 °C using trimethylaluminum and H_2_O precursors.

We synthesized monolayer WSe_2_ on SiO_2_/Si substrates in a 2-in.-diameter quartz tube furnace by using a home-built metal–organic chemical vapor deposition (MOCVD) system. Tungsten hexacarbonyl (THC) and dimethyl selenide (DMSe) were used as precursors for W and Se. Each precursor was kept in its own bubbler at a constant pressure of 800 Torr and transported to the growth chamber through an individual mass flow controller with argon (Ar) as a carrier gas. To improve the WSe_2_ quality, we also introduced hydrogen (H_2_) and oxygen (O_2_) to the growth chamber in order to suppress unwanted carbon residue and assist the precursor decomposition (3–5). Constant flow rates of each gas were used as follows: 360 sccm Ar, 6 sccm THC, 0.6 sccm DMSe, 5 sccm H_2_, and 0.16 sccm O_2_. Sodium chloride was placed upstream of the substrate to increase the growth rate. The WS_2_ flakes were grown at 680 °C for 5 h at a pressure of ∼2 Torr.

The TMD transfer process described in Figure 3a begins by spin coating an 8 wt % PC solution onto the TMD and curing at 150 °C for 10 min. After cooling, TRT is adhered to the stack. The stack is dipped in water before lifting the TRT/PC/TMD stack off the growth substrate to allow water to intercalate beneath the TMD and assist lift-off. The stack is dried and then placed onto the target substrate. The stack is gently annealed at 80 °C for 10 h. Then the temperature is ramped at 5 °C/min to 150 °C, releasing the TRT. The sample is then submerged in a 99.8% CF bath for 2 h followed by a 1 min bath in IPA. The sample is then annealed at 100 °C in a N_2_ glovebox for 30 min.

## Supplementary Material



## Notes

Additional experimental details including upconversion measurement setup, beam size calculation, and far-field vs near-field brightness comparison (PDF)
