# Supplementary material for: Plasmons Enable Ultralow Threshold Solid-State Triplet Fusion Upconversion with a 2D Sensitizer
Source: Nano Lett. 2026 Mar 16;26(12):4220–6. doi: 10.1021/acs.nanolett.6c00279 (PMC13047668; doi:10.1021/acs.nanolett.6c00279)
Supplement: Supplementary file 1 [file nl6c00279_si_001.pdf]

Electronic Supplementary Information

for

Plasmons enable ultralow threshold solid-state triplet fusion upconversion with a 2D sensitizer

Seamus S. Lowe[1], Satya P. Butler[1], John E. Anthony[2], Saien Xie[3,4], Barry P. Rand[3,5]*

[1] Department of Mechanical & Aerospace Engineering
Princeton University
Princeton, NJ, USA, 08544

[2] Department of Chemistry
University of Kentucky
Lexington, KY, USA, 40506

[3] Department of Electrical & Computer Engineering
Princeton University
Princeton, NJ, USA, 08544
Email: brand@princeton.edu

[4] Princeton Materials Institute
Princeton University
Princeton, NJ, USA, 08544

[5] Andlinger Center for Energy and the Environment
Princeton University
Princeton, NJ, USA, 08544

Table of Contents

1. Upconversion measurement setup 2
2. Beam size 3
3. Brightness of near field and far-field emission 4

1. Upconversion measurement setup

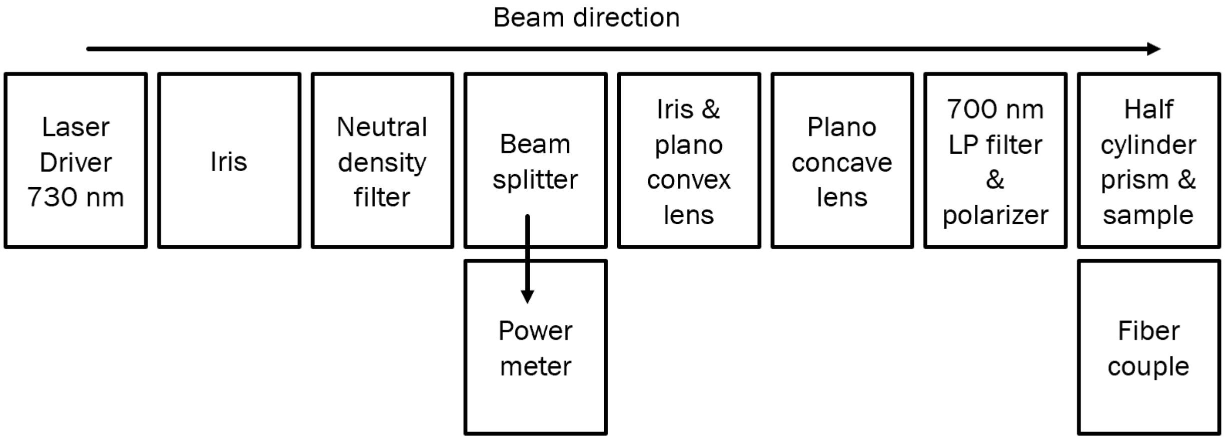

Figure S1: Flow diagram showing optical elements through beam path.

Figure S1 shows a flow diagram of the optical measurement setup used to measure plasmon-enhanced upconversion. We start with a Thorlabs DJ532-40 diode pumped solid state (DPSS) laser which is first passed through an iris to remove stray beams. This is followed by a linear neutral density filter which can be adjusted manually to an optical density of up to 4. The beam then passes through a 90/10 beam splitter, where the weaker beam is directed to a power meter for in-situ power monitoring. The stronger beam passes through a plano-convex lens to focus the beam to a smaller size. A subsequent plano-concave lens collimates the beam.

Next, the beam passes through a rotatable polarizer to make the beam entirely TM-polarized. The beam then passes through a 700 nm long pass (LP) filter to remove any higher energy secondary laser peaks. Finally, the beam reaches a fused silica half cylinder prism (n = 1.461) to which the sample is attached using index matching fluid (n = 1.459). The prism and sample are mounted to a rotation stage along with a fiber couple which leads to the spectrometer.

For far field measurements, the setup is identical. However, before entering the prism, the beam is redirected using three mirrors to hit the sample surface at normal incidence as depicted in **Figure 5b**. Mirror losses are accounted for in far-field calculations.

2. Beam size

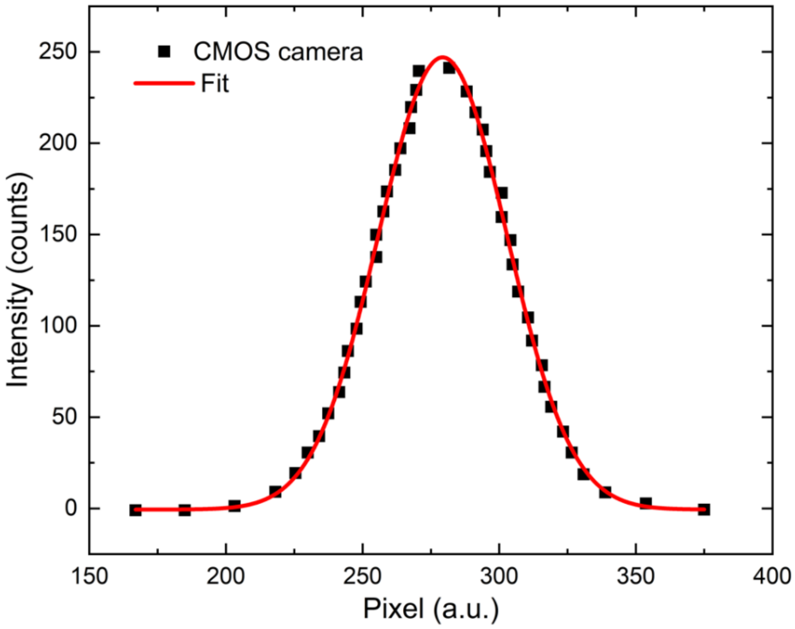

Figure S2: Gaussian fit to beam profile points from CMOS beam capture.

Beam size was measured using a Thorlabs DCC1645C CMOS camera and fit to a Gaussian in horizontal and vertical directions. The 1/e^2 method was used to calculate the horizontal and vertical beam diameters. The beam area is calculated from these diameters. An example of this is shown in **Fig. S2** above.

Furthermore, the beam size is corrected for the angle of incidence on the sample in the Kretschmann configuration. For measured horizontal beam diameter, $d_{m,x}$, and angle of incidence from normal, θ, the horizontal beam diameter on the sample, d_x, is:

$$d_x = \frac{d_{m,x}}{\cos(\theta°)}$$

The vertical beam diameter on the sample is equivalent to the measured vertical diameter.

3. Brightness of near field and far-field emission

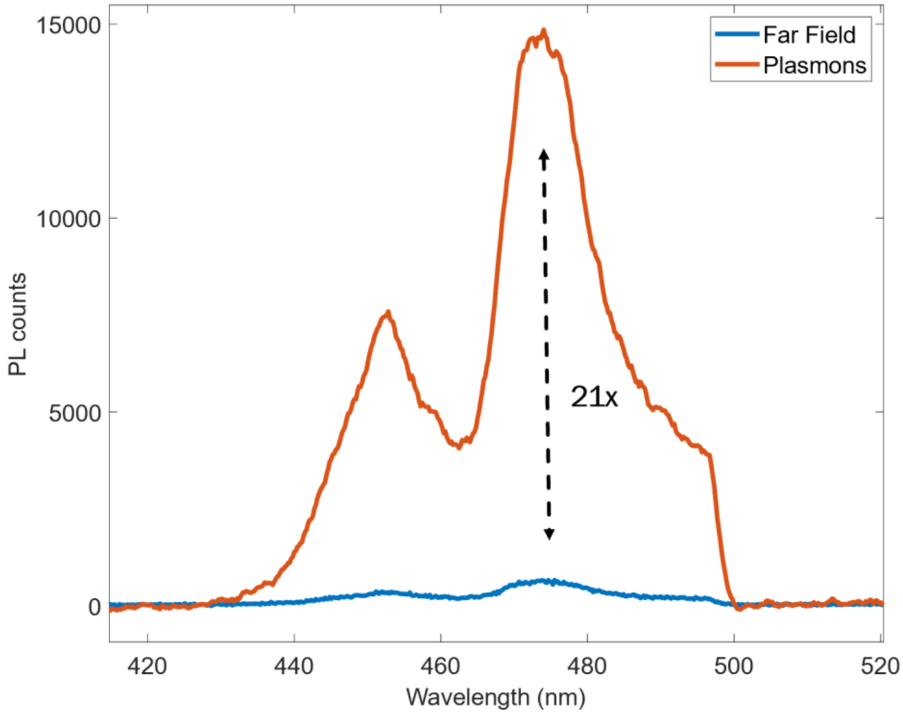

Figure S3: Spectra of anti-Stokes emission for plasmon and far-field excited upconversion.

The brightness of anti-Stokes emission from plasmon-enhanced upconversion is compared to that from far-field excited upconversion in **Figure S3**. As detailed in the manuscript, both systems are operated at similar relative efficiencies to observe an upconversion brightness enhancement of approximately 21x.
